# A Short Message Service Intervention to Support Adherence to Home-Based Strengthening Exercise for People With Knee Osteoarthritis: Intervention Design Applying the Behavior Change Wheel

**DOI:** 10.2196/14619

**Published:** 2019-10-18

**Authors:** Rachel K Nelligan, Rana S Hinman, Lou Atkins, Kim L Bennell

**Affiliations:** 1 Centre for Health Exercise and Sports Medicine Department of Physiotherapy The University of Melbourne Parkville Australia; 2 Centre for Behaviour Change University College London London United Kingdom

**Keywords:** text messaging, mobile phone, knee osteoarthritis, exercise

## Abstract

**Background:**

Knee osteoarthritis is a chronic condition with no known cure. Treatment focuses on symptom management, with exercise recommended as a core component by all clinical practice guidelines. However, long-term adherence to exercise is poor among many people with knee osteoarthritis, which limits its capacity to provide sustained symptom relief. To improve exercise outcomes, scalable interventions that facilitate exercise adherence are needed. SMS (short message service) interventions show promise in health behavior change. The Behavior Change Wheel (BCW) is a widely used framework that provides a structured approach to designing behavior change interventions and has been used extensively in health behavior change intervention design.

**Objective:**

The study aimed to describe the development of, and rationale for, an SMS program to support exercise adherence in people with knee osteoarthritis using the BCW framework.

**Methods:**

The intervention was developed in two phases. Phase 1 involved using the BCW to select the target behavior and associated barriers, facilitators, and behavior change techniques (BCTs). Phase 2 involved design of the program functionality and message library. Messages arranged into a 24-week schedule were provided to an external company to be developed into an automated SMS program.

**Results:**

The target behavior was identified as participation in self-directed home-based strengthening exercise 3 times a week for 24 weeks. A total of 13 barriers and 9 facilitators of the behavior and 20 BCTs were selected to use in the intervention. In addition, 198 SMS text messages were developed and organized into a 24-week automated program that functions by prompting users to self-report the number of home exercise sessions completed each week. Users who reported ≥3 exercise sessions/week (adherent) received positive reinforcement messages. Users who reported &lt;3 exercise sessions/week (nonadherent) were asked to select a barrier (from a list of standardized response options) that best explains why they found performing the exercises challenging in the previous week. This automatically triggers an SMS containing a BCT suggestion relevant to overcoming the selected barrier. Users also received BCT messages to facilitate exercise adherence, irrespective of self-reported adherence.

**Conclusions:**

This study demonstrates application of the BCW to guide development of an automated SMS intervention to support exercise adherence in knee osteoarthritis. Future research is needed to assess whether the intervention improves adherence to the prescribed home-based strengthening exercise.

## Introduction

Knee osteoarthritis is a chronic, highly prevalent condition with no known cure [1] and is a leading contributor to the burden of disease globally [2]. Pain and impaired physical function are characteristic symptoms leading to disability, inactivity, and reduced quality of life [3,4]. Self-management and lifestyle modification to facilitate long-term symptom relief is advocated [5]. This includes exercise, which is recommended in all clinical guidelines irrespective of the person’s age, disease severity, pain, physical dysfunction, and/or comorbidities [5-7]. Unfortunately, adherence to exercise is often poor, particularly in the mid to longer term [8-13], limiting its capacity to provide long-term symptom relief. To improve exercise outcomes, scalable interventions that facilitate exercise adherence are a research priority.

Adherence, defined as the extent to which a person’s behaviour corresponds with agreed recommendations [14], is complex, multifaceted, and not fully understood [11,15,16]. As such, designing interventions to promote exercise adherence is challenging. To aid complex intervention design, the use of theoretical frameworks is recommended [17-19]. The Behavior Change Wheel (BCW) is a synthesis of 19 models of behavior [20,21] that has been used extensively in intervention design [22-27]. When applying the BCW, the first step of intervention design is to use the Capability, Opportunity, Motivation model of behavior (COM-B) to analyze the desired behavior and identify key barriers to and facilitators of the behavior that the intervention is intended to change. The COM-B model describes three interacting categories that influence behavior: (1) capability that includes physical capability (eg, physical skill) and psychological capability (eg, knowledge and psychological skill); (2) opportunity that includes physical opportunity (eg, the environment such as time and resources) and social opportunity (eg, social cues, norms, and interpersonal influences); and (3) motivation including reflective motivation (eg, self-conscious intentions and beliefs) and automatic motivation (eg, emotional reactions, desires, and impulses). These categories can be further divided into the Theoretical Domains Framework (TDF), 14 additional domains of behavior if greater detail is required [21]. Once the behavior has been analyzed using the COM-B model, the BCW then provides recommendations about the functions that interventions could serve to bring about change (eg, education or training) and guides the selection of potential behavior change techniques (BCTs—*active ingredients* designed to bring about change) that could deliver selected intervention functions.

SMS or mobile phone text messaging programs are becoming an increasingly popular delivery method for health behavior change interventions [28-34]. This is unsurprising considering the widespread use of mobile phones across all populations and age groups [29] and the many benefits of using SMS technology. These include instantaneous communication [30], convenience, cost-effectiveness [35], and, most importantly, high user acceptability [36]. Reviews do, however, suggest the need for caution when drawing conclusions about the effectiveness of SMS health behavior change interventions owing to the overall low quality of individual studies [37,38] and the absence of interventions that are rigorously informed by behavior change theory [34,39].

To our knowledge, only two small studies have specifically assessed mobile phone message interventions to support exercise adherence in people with knee osteoarthritis [40,41]. First, a pilot study on 14 participants evaluated 12 video messages (multimedia messaging service [MMS]), delivered every second day to provide visual prompts to exercise [40]. Over the 6-week intervention, there was no change in functional outcomes or exercise adherence. Participants who received the MMS messages did, however, highly value them as reminders. Second, a feasibility study on 27 participants assessed the effects of an educational booklet about knee osteoarthritis and exercise (delivered by mail) plus 4 weekly activity-promoting text messages [41]. Message content was informed by the social cognitive theory, although how exactly the theory informed the intervention design was not described. Significant improvements in pain and exercise self-efficacy were reported. In total, 96% of participants enjoyed receiving the messages and 88% found them useful. Collectively, the limited research to date suggests that people with knee osteoarthritis find SMS technology an acceptable method to support home exercise.

The ability of automated SMS programs to improve adherence to home-based exercise has been demonstrated in healthy adults [42] and adults with frozen shoulder [43]. A randomized controlled trial (RCT) assessed the effect of a 12-week automated SMS program to promote adherence to weekly home-based strengthening exercise in nonexercising healthy adults (aged 50-70 years) from an upper-middle-income country [42]. Participants in the intervention and control received exercise prescription via a booklet. In addition to the exercise booklet, participants in the SMS intervention also received 60 SMS text messages (1 per weekday) containing BCTs aimed to provide encouragement and exercise reminders. BCT selection was informed by the literature. Messages came from a standard set and were not tailored. At 12 weeks, participants receiving the SMS exercised significantly more than participants who received the exercise booklet only. These benefits were not sustained once SMS contact ceased and had diminished by a 24-week follow-up. A second RCT, which assessed the additive effect of a 2-week automated SMS intervention to therapist-prescribed shoulder exercise for people with frozen shoulder, found significantly higher exercise adherence and improved shoulder range of movement in the SMS group compared with the control group who received exercise instruction only [43]. Messages in this study included reminders, encouragement, and education.

To explore if SMS interventions are effective in promoting exercise adherence in knee osteoarthritis, specifically, theory informed and systematically designed interventions that are clearly reported and rigorously assessed are needed. Therefore, the aim of this study was to describe the development of, and rationale for, an SMS behavior change intervention designed by applying the BCW for people with knee osteoarthritis to support exercise adherence.

## Methods

### Overview

The SMS intervention was developed in two phases. Phase 1 applied the BCW framework [20,44] to identify barriers to and facilitators of the target behavior to inform selection of the intervention content. Phase 2 developed the SMS program functionality (ie, how users interact with the program) and the SMS text message library, according to published recommendations for the development of mobile phone text message health behavior interventions [19].

#### Phase 1: Applying the Behavior Change Wheel Framework to Inform Intervention Design

The three stages outlined in the BCW were applied (see Textbox 1) [20,21]. The three stages were initially completed by one author (RN) and then reviewed and discussed by three authors (RN, KB, and RH). The process was overseen by one author (LA), a behavior change expert involved in developing the original BCW framework and with extensive experience in its practical application.

Stage 1 involved gaining a thorough understanding of the behavior. Through reviewing the literature, the problem was defined in behavioral terms and the target behavior selected and explained in detail with context. To identify what needs to change to support the target behavior, we drew on the findings of a scoping review previously conducted by members of our team [45]. This review synthesized key barriers and facilitators for people with hip and/or knee osteoarthritis to participate in intentional exercise, mapped according to the TDF domains. Barriers and facilitators relevant to the target behavior and suitable for SMS delivery were selected from this review and organized according to the COM-B model of behavior.

Stages 2 and 3 used the BCW mapping process to select intervention functions and BCTs to address the barriers and facilitators identified in Stage 1 and with the capacity to be incorporated into the SMS format. First, this involved selecting intervention functions appropriate for SMS delivery that link to the COM-B categories identified in Stage 1. Intervention functions are the types of interventions most likely to bring about change in behavior and include education, persuasion, incentivisation, coercion, training, restriction environmental restructuring, modelling, and enablement. Once intervention functions were selected, BCTs relevant to each intervention function were chosen from the BCT Taxonomy (BCTTv1) [46].

**Stages of intervention design using the behavior change technique.**

1. Understand the behavior
  1. Define the problem in behavioral terms
  2. Select target behavior
  3. Specify the target behavior
  4. Identify what needs to change
2. Identify intervention options
  1. Identify intervention functions
  2. Identify policy categories
3. Identify content and implementation options
  1. Identify behavior change techniques
  2. Identify mode of delivery

#### Phase 2: Development of the SMS Program Functionality and Message Library

Construction of the SMS program functionality (including message type, message frequency, and level of program interaction) was guided by published recommendations [19] and the associated literature. To develop the message library, BCTs linked to the barriers and facilitators identified in phase 1 were converted into an SMS of a maximum 308 characters. A total of 12 people (7 academics working in knee osteoarthritis conservative management, 4 clinical physiotherapists, and 1 person with knee osteoarthritis) individually provided input about message wording. Three authors (RN, RH, and KB) compiled this feedback and used it to construct the final SMS library. Samples of messages were reviewed by one author (LA) to ensure SMS wording accurately reflected the BCW mapping process (from phase 1) and the intended BCT. The final SMS library was then arranged into a 24-week schedule. The literacy demands of all messages were assessed with Web-based readability software (Readable.io, Added Bytes, Ltd), using the Flesch-Kincaid Grade Level, a mathematical calculation to determine the US grade reading level based on word and sentence length. This is a recommended tool to assist health material writing [47] used previously in the literature [48,49]. A SMS practice sequence was also developed to give users the option to practice replying to messages before commencing the 24-week program. The 24-week message schedule and practice sequence were provided to an external company (SMS Solutions PTY LTD) who were responsible for developing the automated text message program and administrator interface. The 24-week SMS program was trialed by one researcher (RN) and company staff to test functionality and identify message errors.

## Results

### Phase 1: Applying the Behavior Change Technique Framework to Inform Intervention Design

#### Stage 1: Understand the Behavior

The problem was identified as lack of adherence to self-directed home exercise. Home-based exercise in knee osteoarthritis typically includes knee strengthening exercise that is prescribed and supervised by a clinician, such as a physiotherapist. After initial supervision, strengthening exercise is then continued unsupervised by a patient in their home. Home programs are reported to have similar symptomatic effects as supervised exercise (individual or group-based) [50,51]; however, adherence to these programs once supervision ceases often declines [16]. To be effective, home-based strengthening programs should follow strength-based protocols [52]. Adherence over the longer term is required to allow muscle adaptation and habit formation to occur. Habit formation, which can take an average of 2 months and up to 8 months, is a key component to the successful adoption of a new behavior [53] and is particularly important as exercise is encouraged on an ongoing basis for individuals with knee osteoarthritis to ensure long-term symptomatic relief [54]. The target behavior was therefore selected to be participation in structured, self-directed progressive home-based strengthening exercises, 3 times a week. Conservatively, a duration of 24 weeks was selected to facilitate habit formation. The target behavior is described in detail in Table 1.

A total of 9 facilitators and 13 barriers to the target behavior were selected from those identified in the extensive scoping review [45] and considered appropriate for an SMS intervention (Textboxes 2 and 3). These were coded using COM-B/TDF domains and listed in BCW intervention mapping tables (Multimedia Appendices 1 and 2). Barriers within the same COM-B category that had a similar meaning were grouped, resulting in 8 barrier categories. COM-B/TDF mapping as outlined by Dobson et al [45] was retained except for barrier *too tired* with all authors identifying this as a reflective motivation barrier in addition to a psychological capability barrier. From the BCW intervention mapping tables, the COM-B categories of psychological capability, reflective motivation, automatic motivation, and social opportunity were identified as the key areas requiring change to support the target behavior. Most barriers were linked to reflective motivation. Most facilitators were linked to physiological capability.

**Table 1:** Target behavior described in detail.

Target behavior	Participation in a structured, self-directed progressive home-based strengthening exercise, 3 times a week, for 24 weeks
Who needs to perform the behavior?	Individuals with symptomatic knee osteoarthritis
When will they do it?	When convenient to the person with knee osteoarthritis
Where will they do it?	Home-base
How often will they do it?	3 times a week in accordance with exercise guidelines [55] for 24 weeks
With whom will they do it?	Independently

**Facilitators selected as key to participation in structured, progressive strength-based home exercise with the SMS intervention.**

Selected facilitators

1. Accurate osteoarthritis disease knowledge
2. Prioritizing exercise
3. Integrating exercise into daily tasks
4. Belief that you are taking control of your own disability
5. Perceived benefits of exercising
6. Belief that exercise is good for health
7. Positive outcome expectations
8. Long- & Short-term goals
9. Receiving medical advice to exercise

**Barriers selected as key to participation in structured, progressive strength-based home exercise with the SMS intervention.**

Selected barriers

1. Forgetfulness
2. Too tired
3. Knee pain limiting perceived ability to exercise
4. Concerned exercise is causing pain/damage (includes concern exercise is causing pain + fear of damaging knee further)
5. Lack of improvement with exercises
6. Boredom with exercise (includes lack of enjoyment in exercise + boredom with exercise)
7. Lacking time (includes conflict with routines + lack of time)
8. Life stress (includes family commitments + increased social strain + life events)

#### Stage 2: Identifying Intervention Options

A total of five intervention functions were selected: (1) education, (2) persuasion, (3) training, (4) environmental restructuring, and (5) enablement. The five selected intervention functions were added to the BCW intervention mapping tables (Multimedia Appendices 1 and 2).

#### Stage 3: Identifying Content and Implementation Options

To address the 8 barrier categories, 19 BCTs were selected. To address the 9 facilitators, 4 BCTs were selected. Findings from the literature [56-58] were used to reduce the number of possible facilitator BCTs as less facilitator BCT messages were required for the program (refer to Phase 2: Development of how the SMS program functions and the message library). Three BCTs addressed both barriers and facilitators. The BCTs were then added to BCW intervention mapping tables (Multimedia Appendices 1 and 2).

SMS was selected by the authors (KB and RH) as the mode of delivery for an intervention to support exercise adherence at the outset of intervention design. This was based on the available literature identifying SMS as a scalable, effective, efficient, and affordable tool to promote adherence to a range of health behaviors including physical activity and exercise [29-33,59,60]. This decision limited barriers, facilitators, intervention function, and BCT selection to those who would be suitable to be addressed via SMS.

### Phase 2: Development of How the SMS Program Functions and the Message Library

The SMS program was developed to incorporate the barriers and facilitators and BCTs identified in phase 1 and be automated, interactive, and tailored, important characteristics of effective SMS behavior change interventions [19,31].

**Figure 1:**
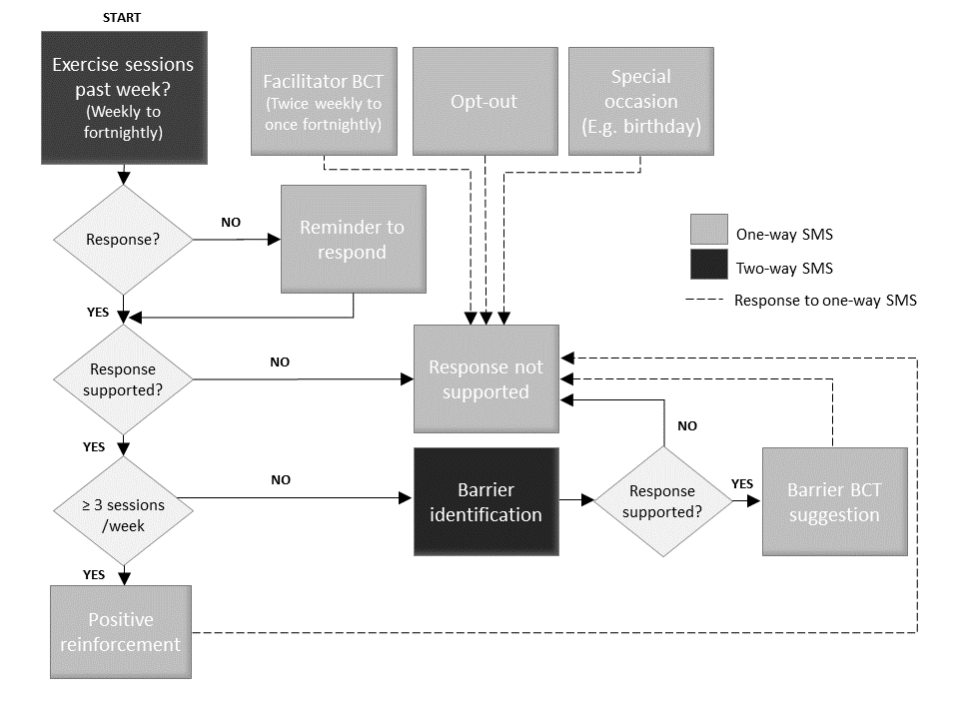
Message interactions and triggers. BCT= behavior change technique.

A total of 9 message types were developed (see Multimedia Appendices 3 and 4) for the automated SMS program. The automated SMS sequence was designed to start with an initial message asking the participant to self-report the number of home exercise sessions they completed in the previous week. Users who self-reported ≥3 exercise sessions/week (adherent) were sent a positive reinforcement message aimed to encourage continued adherence. Users who reported <3 exercise sessions/week (nonadherent) were sent a message asking them to reply by selecting one barrier (from a predefined list of 9 options, including an option of *none above apply to me*) which best explains what made doing the prescribed exercises in the previous week challenging. The user’s reply triggers an SMS response containing a BCT suggestion relevant to their selected barrier. BCT messages to facilitate exercise adherence are also sent, irrespective of self-reported adherence. Figure 1 provides a diagrammatic representation of message interactions and triggers.

To address user messages that are not recognized by the program automation (eg, replies to one-way messages or replies not from a predefined list), a *response not supported* message was created encouraging the user to try again or contact program staff if needed. Where appropriate, messages were designed to contain participants’ first names. To assist program use (eg, replying to messages), a guided three-message practice sequence was developed. The practice sequence is activated by sending the word START to the phone number assigned to the SMS program.

Guidance from the literature regarding message frequency is highly varied, ranging from daily to once weekly [29,30,33,59], although it has been recommended that 3 messages per week are suitable, with more messages sent initially [19]. Message frequencies that decrease over time appear to be more effective [19,31]. In line with these recommendations, the SMS program was designed to send 4 to 5 messages per week and be reduced to 2 per week by completion of the intervention. Multimedia Appendices 3 and 4 outline message frequency, and the number of each message type required for the 24-week intervention period.

On the basis of how the program automation works and the message frequency, 198 messages were developed for the intervention: 144 barrier BCT messages (16 per barrier category plus 16 messages relating to *none apply to me*); 24 facilitator BCT messages; 20 positive reinforcement messages; and 10 program logistic messages. A total of 144 barrier messages were required to ensure users do not receive duplicate barrier BCT messages in the event that the same barrier is selected more than once over the 24-week intervention. The literacy demands of all messages in the 24-week schedule were assessed as 5.4 grade, well below the maximum 8th-grade reading level recommended for consumer health care information [19,61]. All messages were arranged into a 24-week schedule, ensuring a spread of BCTs within each COM-B category.

**Figure 2:**
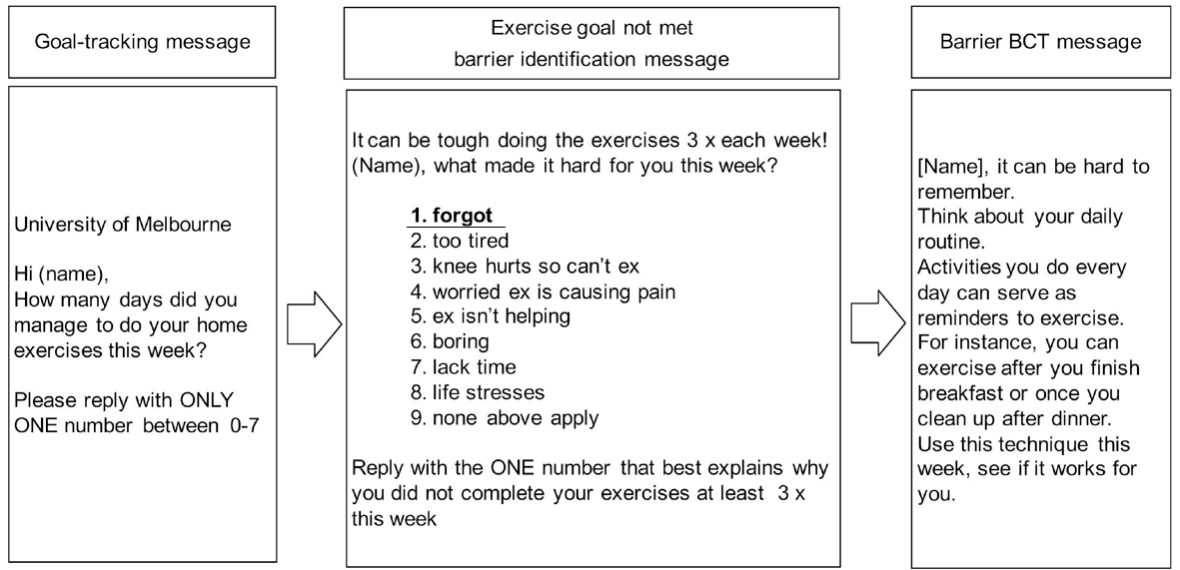
Example automated message sequence for low exercise adherence (<3 exercise sessions) and barrier ‘forgot'. BCT= behavior change technique.

Figure 2 provides an example of an automated message sequence for reported low exercise adherence. Tables 2 and 3 provide examples of the mapping of barrier and facilitator BCT messages following the BCW framework.

The 24-week message schedule and practice sequence was provided to SMS Solutions PTY LTD who were contracted to convert both into an automated text message program with administrative access via a password-protected website. SMS Solutions PTY LTD provided training to the research staff in program and website interface use. The program was then pretested with minor spelling and message duplication errors identified and corrected. The website interface enables addition of program users by entering their name, mobile phone number, birth date, and program start date (set as the following Monday) which triggers the automated 24-week message sequence. The sequence commences with a *Goal Tracking* message. All user messages sent and received are recorded within the program website. All unsupported user communication (eg, replies to one-way messages) is marked with an identifier to allow easy monitoring and subsequent follow-up by research staff if required.

**Table 2:** Example mapping of barrier behavior change technique messages following the behavior change wheel framework.

Barrier	COM-B^a^ category	TDF^b^ domain	Intervention function	BCT^c^	SMS^d^ content
Forgetfulness	Psychological capability	10. Memory, attention and decision processes	Training	8.3 Habit formation	[Name], it can be hard to remember. We suggest making the exercises a habit. Set aside the same time each day to do them. It’s much harder to forget when something is a daily routine.
Concern exercise (causing) pain	Reflective motivation	6. Beliefs about consequences	Enablement	1.2 Problem Solving	Remember mild pain with knee exercise is okay but significant pain’s discouraging. Figure out the cause! Use a log book this week, record how you feel after EACH exercise. Identify the exercise linked to your concern. Modify that ONE exercise & keep doing your program 3x week.

^a^COM-B: Capability, Opportunity, Motivation model of behavior.

^b^TDF: Theoretical Domains Framework.

^c^BCT: behavior change technique.

**Table 3:** Example mapping of facilitator behavior change technique messages following the behavior change wheel framework. OA: osteoarthritis.

Facilitator	COM-B^a^ category	TDF^b^ domain	Intervention function	BCT^c^	SMS^d^ content
Accurate disease knowledge	Psychological capability	1. Knowledge	Education	5.1 Information about health consequences	Let’s bust this myth - Surgery is not inevitable if you have knee OA! Exercise is one of the most effective ways to reduce your knee pain and prevent surgery. Stick with your exercise to see the benefits!
Prioritizing exercise	Psychological capability	14. Behavioral regulation	Enablement	10.9 Self reward	Did you prioritize your exercise this week and get them done? Then reward yourself, (name)! Sticking to an exercise program for this long is a real accomplishment that deserves celebration.

^a^COM-B: Capability, Opportunity, Motivation model of behavior.

^b^TDF: Theoretical Domains Framework.

^c^BCT: behavior change technique.

## Discussion

### Overview

This study reports the systematic design of an intervention to support exercise adherence targeting people with knee osteoarthritis using the BCW framework and current evidence. The intervention is a 24-week automated, semipersonalized mobile phone SMS program to support adherence to prescribed home-based strengthening exercise 3 times a week. The program aims to do this by asking users how many home exercise sessions they completed in the previous week, providing positive reinforcement messages to adherent users, prompting barrier identification and providing targeted behavior change support messages to nonadherent users, and providing facilitation to exercise messages irrespective of weekly exercise adherence.

### Strengths and Limitations

The systematic design of the SMS intervention content is a significant strength of this study. Application of the BCW provided a clear framework to develop a targeted and informed intervention that incorporates BCTs specifically selected to address known barriers and facilitators to exercise adherence in people with knee osteoarthritis. The BCW also provided structure for the transparent and thorough reporting of intervention design, vital for future evidence synthesis that will enable a greater understanding of how digital behavior change interventions may have their effect [44]. There are several additional strengths related to the SMS program functionality. Owing to its automated design, minimal administrative support is required throughout the 24-week intervention, making it scalable and potentially cost-effective for both the deliverer and user. The program includes weekly to fortnightly exercise monitoring and provides instantaneous feedback, important characteristics of effective exercise adherence interventions [13,44]. The use of keywords selected from predetermined lists triggers appropriate replies and enables tailoring of content, important in addressing users’ individual needs. Intervention fidelity is also guaranteed with all users receiving standardized evidence-based information.

Several limitations should be acknowledged. First, the SMS delivery mode requires users to have adequate vision, a reasonable level of English literacy, access to a mobile phone, and ability to use the SMS function on their phone. However, 87% of Australian adults aged above 55 years use a mobile phone [62] and 75% of all mobile phone users are regular text message senders [63], suggesting this SMS intervention is broadly relevant to most Australians. Second, users who report low exercise adherence must select from a predetermined list of barrier categories that may not specifically target their unique needs. Users are also not able to seek clarification of message content and do not receive additional support that may be required if the same barrier is selected repeatedly. The program functionality does, however, ensure a user will not receive the same BCT message in response to selecting the same barrier. For future implementation of this SMS program, several factors must be considered such as funding sources for intervention delivery (approximately Aus $8 for the 24-week outgoing messages per user) and the need for some level of ongoing administrative support to follow up emergency messages, if received.

### Future Research

We intend to evaluate the feasibility, effectiveness, and acceptability of the SMS program to support adherence to prescribed home-based strengthening exercise in RCTs, using both quantitative and qualitative methods. If effective, the intervention could be an easily scalable, cost-effective, convenient solution to support adherence to prescribed home-based strengthening exercise for people with knee osteoarthritis. The intervention could be incorporated into current or future exercise resources (eg, Web-based, remotely delivered exercise programs) or be provided by health professionals to facilitate exercise adherence, improve exercise outcomes, and ultimately improve longer term knee osteoarthritis symptom management. The program could also be adapted for use in other health conditions where exercise adherence is needed.

### Conclusions

This study describes how the BCW can be successfully and systematically used to guide development of an automated SMS intervention to support exercise adherence in knee osteoarthritis. Future research is needed to assess whether the intervention improves adherence to prescribed home-based strengthening exercise and is accepted by users, people with knee osteoarthritis.

## Appendix

Multimedia Appendix 1 Mapping barrier to exercise to behavior change technique using the Behavior Change Wheel.

Multimedia Appendix 2 Mapping facilitator to exercise to behavior change technique using the Behavior Change Wheel.

Multimedia Appendix 3 Description and frequency of behavior change automated messages.

Multimedia Appendix 4 Description and frequency of logistic and other automated messages.

## Abbreviations

BCT: behavior change technique
BCW: Behavior Change Wheel
COM-B: Capability, Opportunity, Motivation model of behavior
MMS: multimedia messaging service
RCT: randomized controlled trial
TDF: Theoretical Domains Framework
