# Supplementary material for: A Short Message Service Intervention to Support Adherence to Home-Based Strengthening Exercise for People With Knee Osteoarthritis: Intervention Design Applying the Behavior Change Wheel
Source: JMIR Mhealth Uhealth. 2019 Oct 18;7(10):e14619. doi: 10.2196/14619 (PMC7012505; doi:10.2196/14619)
Supplement: Multimedia Appendix 1 [file mhealth_v7i10e14619_app1.pdf]

Barrier selected	COM-B category	TDF Domain	Relevant intervention function	BCT used within the SMS intervention from BCTTv1
Forgetfulness	Psychological capability	10. Memory, attention and decision processes	Training	8.3 Habit formation
			Environmental restructuring	7.1 Prompts/cues
			Enablement	3.1 Social support (unspecified)
				10.9 Self-reward
				1.2 Problem Solving
				1.4 Action Planning
Too tired	Psychological capability	10. Memory, attention and decision processes	Education*	5.1 Information about health consequences *
			Persuasion*	-
	Reflective motivation*	8. Intentions	Training	-
			Environmental restructuring	-
			Enablement	1.4 Action planning
				15.4 Self talk
				15.1 Verbal persuasion about capability
				1.2 Problem Solving
				8.7 Graded tasks
Knee pain limiting perceived ability to exercise	Reflective motivation	4. Beliefs about Capabilities	Education	5.1 Information about health consequences
			Persuasion	-
			Enablement	8.7 Graded tasks
				12.4 Distraction
				15.4 Self talk
				1.4 Action Planning
				1.2 Problem Solving
Concern exercise	Reflective motivation	6. Beliefs about consequences	Persuasion	5.1 Information about health consequences
			Enablement	1.2 Problem Solving

(causing) pain + Fear of damaging knee further				1.4 Action planning
Lack of improvement with exercises	Automatic motivation	7. Reinforcement	Training	2.4 Self-monitoring outcome(s) of behaviour
				2.2 Feedback on behaviour
				4.4 Behavioural experiments
				4.1 Instruction on how to perform the behaviour
			Environmental restructuring	-
Lack of enjoyment in exercise + Boredom with exercise	Automatic motivation	13. Emotion	Persuasion	5.1 Information about health consequences
			Enablement	12.4 Distraction
				15.4 Self talk
				10.9 Self-reward
				12.1 Restructuring the physical environment
				1.2 Problem Solving
				1.4 Action Planning
				3.1 Social support (unspecified)
Conflict with routines + Lack of time	Reflective motivation	11. Environmental context and resources	Training	4.1 Instruction on how to perform the behaviour
			Environmental restructuring	-
			Enablement	3.1 Social support (unspecified)
				1.2 Problem solving
				1.4 Action Planning
				15.3 Focus on past success

				1.3 Goal setting (outcome)
Family commitments<br>+<br>Increased social strain<br>+<br>Life events	Social Opportunity	12. Social influences	Environmental restructuring	-
			Enablement	1.4 Action Planning
				2.4 Self-monitoring of outcome(s) of behaviour
				15.1 Verbal persuasion about capability
				1.2 Problem solving
				15.4 Self talk
				15.3 Focus on past success
				1.1 Goal setting (behaviour)

Dash (-) = no BCT message used in final SMS library from this intervention function

BCT= Behavior change technique; BCW=Behavior Change Wheel;

COM-B= Capability, Opportunity, Motivation model of behavior;

BCTTv1= Behavior change technique taxonomy
