# Supplementary material for: A Short Message Service Intervention to Support Adherence to Home-Based Strengthening Exercise for People With Knee Osteoarthritis: Intervention Design Applying the Behavior Change Wheel
Source: JMIR Mhealth Uhealth. 2019 Oct 18;7(10):e14619. doi: 10.2196/14619 (PMC7012505; doi:10.2196/14619)
Supplement: Multimedia Appendix 2 [file mhealth_v7i10e14619_app2.pdf]

Facilitators	COM-B category	TDF	Intervention Function	BCT used within the SMS intervention from BCTTv1
Accurate disease knowledge	Psychological capability	1. Knowledge	Education	5.1 Information about health consequences
Prioritising exercise	Psychological capability	14. Behavioural regulation	Education	-
			Training	4.1 Instruction on how to perform the behaviour
			Enablement	10.9 Self reward
Integrating exercise into daily tasks	Psychological capability	14. Behavioural regulation	Education	5.1 Information about health consequences
			Training	4.1 Instruction on how to perform the behaviour
			Enablement	-
Belief that you are taking control of own disability	Psychological capability	4. Beliefs about Capabilities	Education	5.1 Information about health consequences
			Persuasion	5.1 Information about health consequences
				6.2 Social Comparison
Perceived benefits of exercising	Psychological capability	6. Beliefs about consequences	Education	5.1 Information about health consequences
			Persuasion	-
Belief that exercise is good for health	Psychological capability	6. Beliefs about consequences	Education	-
			Persuasion	5.1 Information about health consequences
Positive outcome expectations	Psychological capability	6. Beliefs about consequences	Education	-
			Persuasion	5.1 Information about health consequences
				6.2 Social Comparison
Long-term goals as well as short-term goals	Psychological capability	9. Goals	Education	-
			Persuasion	6.2 Social Comparison
			Enablement	10.9 Self reward

Receiving medical advice to exercise	Automatic motivation	7. Reinforcement	Training	4.1 Instruction on how to perform the behaviour
			Environmental restructuring	-

Dash (-) = no BCT message used in final SMS library from this intervention function

BCT= Behavior change technique; BCW=Behavior Change Wheel;

COM-B= Capability, Opportunity, Motivation model of behavior;

BCTTv1= Behavior change technique taxonomy
