# Supplementary material for: A Short Message Service Intervention to Support Adherence to Home-Based Strengthening Exercise for People With Knee Osteoarthritis: Intervention Design Applying the Behavior Change Wheel
Source: JMIR Mhealth Uhealth. 2019 Oct 18;7(10):e14619. doi: 10.2196/14619 (PMC7012505; doi:10.2196/14619)
Supplement: Multimedia Appendix 3 [file mhealth_v7i10e14619_app3.pptx]

## Slide 1
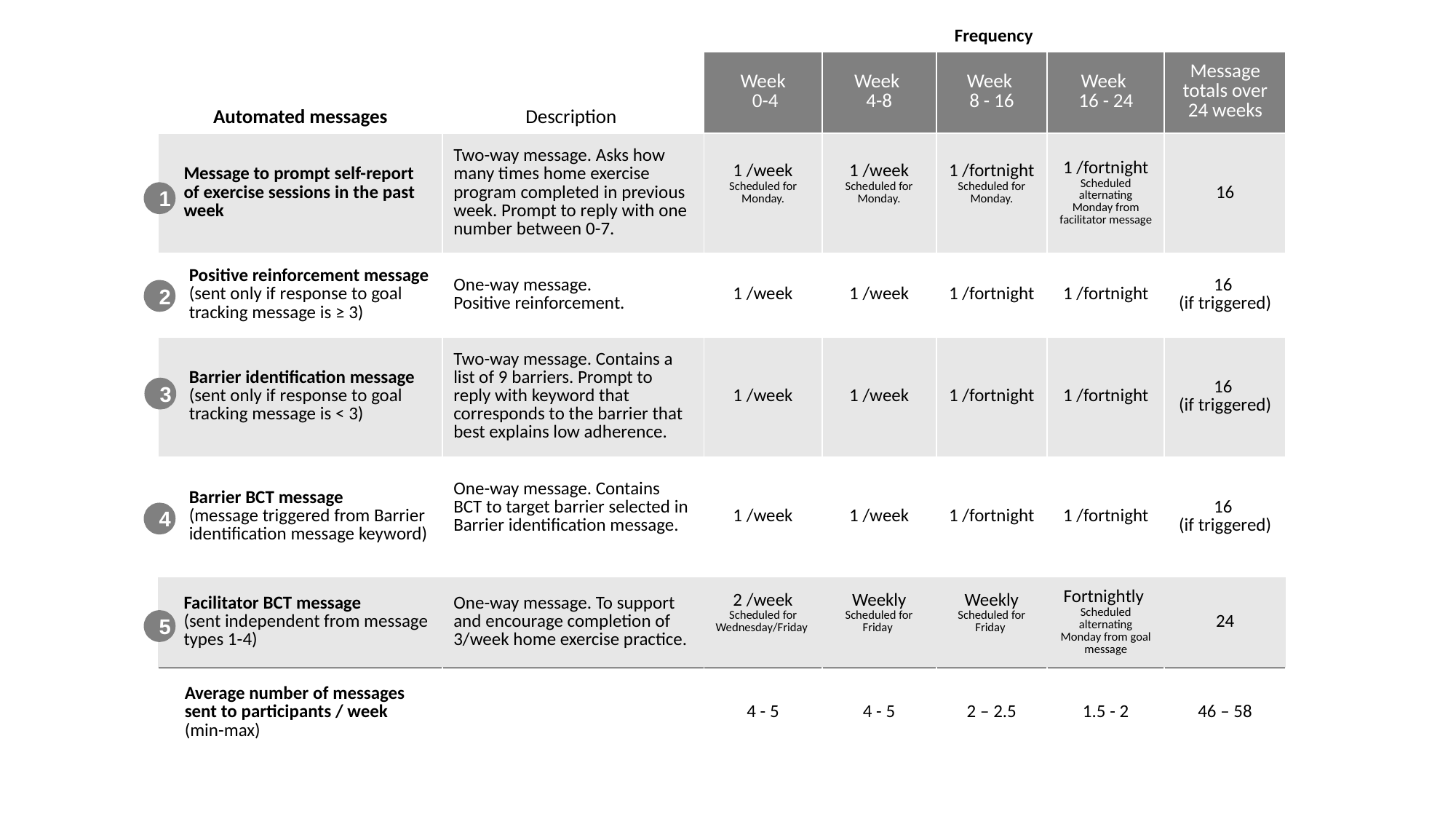

Frequency
| Automated messages | Description | Week 0-4 | Week 4-8 | Week 8 - 16 | Week 16 - 24 | Message totals over 24 weeks |
| --- | --- | --- | --- | --- | --- | --- |
| Message to prompt self-report of exercise sessions in the past week | Two-way message. Asks how many times home exercise program completed in previous week. Prompt to reply with one number between 0-7. | 1 /week Scheduled for Monday. | 1 /week Scheduled for Monday. | 1 /fortnight Scheduled for Monday. | 1 /fortnight Scheduled alternating Monday from facilitator message | 16 |
| Positive reinforcement message (sent only if response to goal tracking message is ≥ 3) | One-way message. Positive reinforcement. | 1 /week | 1 /week | 1 /fortnight | 1 /fortnight | 16 (if triggered) |
| Barrier identification message (sent only if response to goal tracking message is < 3) | Two-way message. Contains a list of 9 barriers. Prompt to reply with keyword that corresponds to the barrier that best explains low adherence. | 1 /week | 1 /week | 1 /fortnight | 1 /fortnight | 16 (if triggered) |
| Barrier BCT message (message triggered from Barrier identification message keyword) | One-way message. Contains BCT to target barrier selected in Barrier identification message. | 1 /week | 1 /week | 1 /fortnight | 1 /fortnight | 16 (if triggered) |
| Facilitator BCT message (sent independent from message types 1-4) | One-way message. To support and encourage completion of 3/week home exercise practice. | 2 /week Scheduled for Wednesday/Friday | Weekly Scheduled for Friday | Weekly Scheduled for Friday | Fortnightly Scheduled alternating Monday from goal message | 24 |
| Average number of messages sent to participants / week (min-max) | | 4 - 5 | 4 - 5 | 2 – 2.5 | 1.5 - 2 | 46 – 58 |
1
2
3
4
5
