# Supplementary material for: A Short Message Service Intervention to Support Adherence to Home-Based Strengthening Exercise for People With Knee Osteoarthritis: Intervention Design Applying the Behavior Change Wheel
Source: JMIR Mhealth Uhealth. 2019 Oct 18;7(10):e14619. doi: 10.2196/14619 (PMC7012505; doi:10.2196/14619)
Supplement: Multimedia Appendix 4 [file mhealth_v7i10e14619_app4.pptx]

## Slide 1
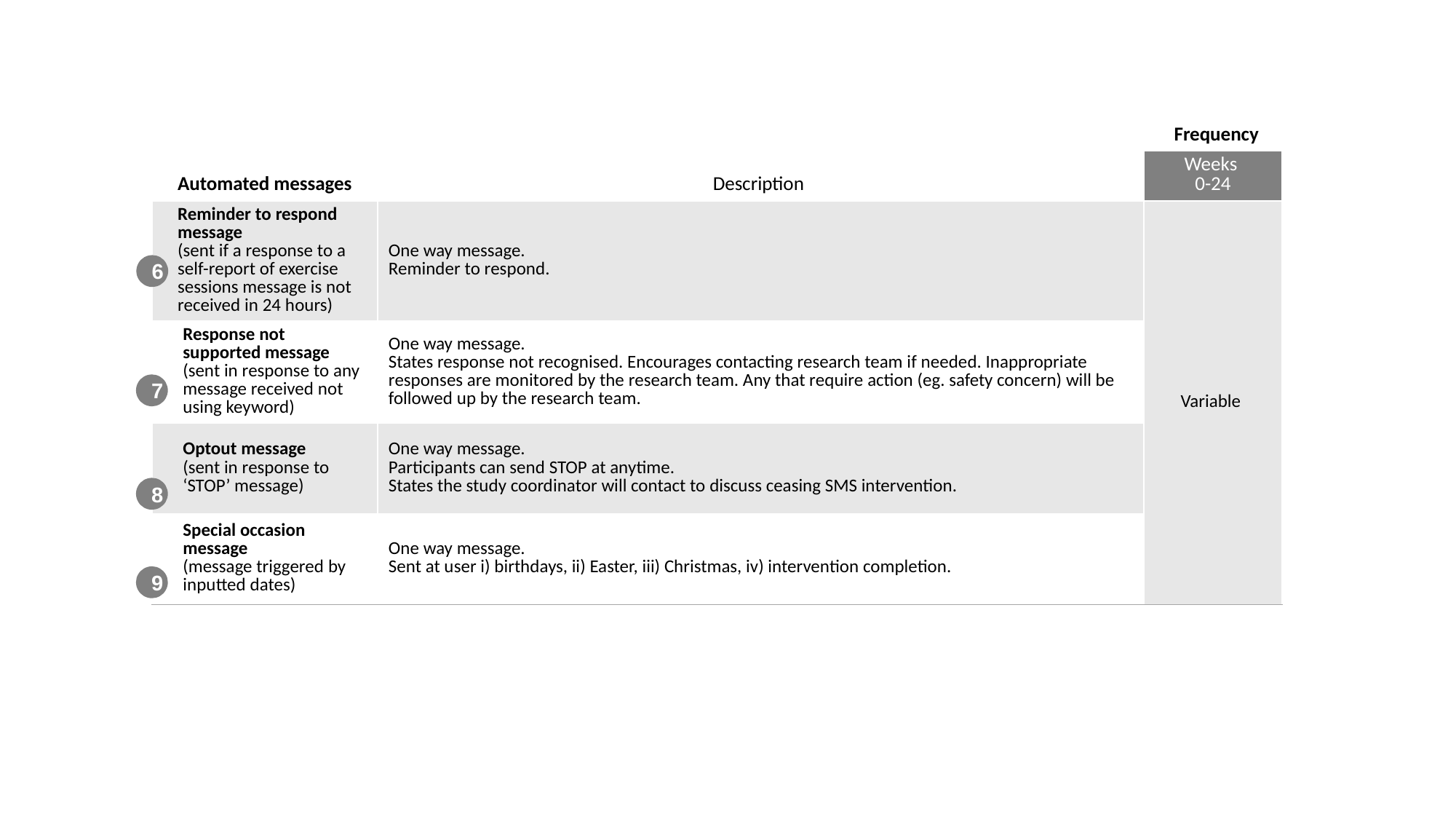

Frequency
| Automated messages | Description | Weeks 0-24 |
| --- | --- | --- |
| Reminder to respond message (sent if a response to a self-report of exercise sessions message is not received in 24 hours) | One way message. Reminder to respond. | Variable |
| Response not supported message (sent in response to any message received not using keyword) | One way message. States response not recognised. Encourages contacting research team if needed. Inappropriate responses are monitored by the research team. Any that require action (eg. safety concern) will be followed up by the research team. | |
| Optout message (sent in response to ‘STOP’ message) | One way message. Participants can send STOP at anytime. States the study coordinator will contact to discuss ceasing SMS intervention. | |
| Special occasion message (message triggered by inputted dates) | One way message. Sent at user i) birthdays, ii) Easter, iii) Christmas, iv) intervention completion. | |
6
7
8
9
